# Supplementary material for: A fault-intrusion-tolerant system and deadline-aware algorithm for scheduling scientific workflow in the cloud
Source: PeerJ Comput Sci. 2021 Nov 2;7:e747. doi: 10.7717/peerj-cs.747 (PMC8576554; doi:10.7717/peerj-cs.747)
Supplement: Supplemental Information 6 [file peerj-cs-07-747-s006.zip › Myproject1.1/docs/org/workflowsim/planning/package-summary.html]

org.workflowsim.planning


JavaScript is disabled on your browser.


- Overview
- Package
- Class
- Use
- Tree
- Deprecated
- Index
- Help

- Prev Package
- Next Package

- Frames
- No Frames

- All Classes

# Package org.workflowsim.planning

- Interface Summary

  | Interface | Description |
  | --- | --- |
  |  |  |
  | --- | --- |
  | PlannerInterface | The Planner interface |
- Class Summary

  | Class | Description |
  | --- | --- |
  |  |  |
  | --- | --- |
  | BasePlanner | The base planner has implemented the basic features. |
  | RandomPlanner | The Random planning algorithm. |

- Overview
- Package
- Class
- Use
- Tree
- Deprecated
- Index
- Help

- Prev Package
- Next Package

- Frames
- No Frames

- All Classes
